# Microbiome Search Engine 2: a Platform for Taxonomic and Functional Search of Global Microbiomes on the Whole-Microbiome Level

**DOI:** 10.1128/mSystems.00943-20

**Published:** 2021-01-19

**Authors:** Gongchao Jing, Lu Liu, Zengbin Wang, Yufeng Zhang, Li Qian, Chunxiao Gao, Meng Zhang, Min Li, Zhenkun Zhang, Xiaohan Liu, Jian Xu, Xiaoquan Su

**Affiliations:** a College of Computer Science and Technology, Qingdao University, Qingdao, China; b Single-Cell Center, CAS Key Laboratory of Biofuels and Shandong Key Laboratory of Energy Genetics, Qingdao Institute of BioEnergy and Bioprocess Technology, Chinese Academy of Sciences, Qingdao, China; c Shandong Institute of Energy Research, Qingdao, China; d National Science Library, Chinese Academy of Sciences, Beijing, China; e Department of Computer Science and Technology, Ocean University of China, Qingdao, China; f Key Laboratory of Marine Drugs, the Ministry of Education of China, School of Medicine and Pharmacy, Ocean University of China, Qingdao, China; Dalhousie University

**Keywords:** amplicon, metagenome, microbiome, online service, search engine

## Abstract

Metagenomic data sets from diverse environments have been growing rapidly. To ensure accessibility and reusability, tools that quickly and informatively correlate new microbiomes with existing ones are in demand. Here, we introduce Microbiome Search Engine 2 (MSE 2), a microbiome database platform for searching query microbiomes in the global metagenome data space based on the taxonomic or functional similarity of a whole microbiome to those in the database. MSE 2 consists of (i) a well-organized and regularly updated microbiome database that currently contains over 250,000 metagenomic shotgun and 16S rRNA gene amplicon samples associated with unified metadata collected from 798 studies, (ii) an enhanced search engine that enables real-time and fast (<0.5 s per query) searches against the entire database for best-matched microbiomes using overall taxonomic or functional profiles, and (iii) a Web-based graphical user interface for user-friendly searching, data browsing, and tutoring. MSE 2 is freely accessible via http://mse.ac.cn. For standalone searches of customized microbiome databases, the kernel of the MSE 2 search engine is provided at GitHub (https://github.com/qibebt-bioinfo/meta-storms).

**IMPORTANCE** A search-based strategy is useful for large-scale mining of microbiome data sets, such as a bird’s-eye view of the microbiome data space and disease diagnosis via microbiome big data. Here, we introduce Microbiome Search Engine 2 (MSE 2), a microbiome database platform for searching query microbiomes against the existing microbiome data sets on the basis of their similarity in taxonomic structure or functional profile. Key improvements include database extension, data compatibility, a search engine kernel, and a user interface. The new ability to search the microbiome space via functional similarity greatly expands the scope of search-based mining of the microbiome big data.

## Abstract (precis)

A search-based strategy is useful for large-scale mining of microbiome data sets, such as a bird’s-eye view of the microbiome data space and disease diagnosis via microbiome big data. Here, we introduce Microbiome Search Engine 2 (MSE 2), a microbiome database platform for searching query microbiomes against the existing microbiome data sets on the basis of their similarity in taxonomic structure or functional profile.

## INTRODUCTION

Metagenomic approaches have been widely employed to probe microbiomes among various habitats by linking dynamics of microbial compositions and predicted functions to environmental changes (1, 2), human disease development (3–7), and drug responses (8, 9). With the rapid development of sampling strategies and sequencing technologies, an enormous volume of microbiome data sets, including both 16S rRNA gene amplicon-based and shotgun whole-genome sequencing (WGS)-based data sets, has been produced by individual, small-cohort projects or large-scale surveys, such as the Human Microbiome Project (10), the Earth Microbiome Project (11), the American Gut Project (12), and Tara Oceans (13). Most DNA sequence data are stored in either general-purpose DNA sequence repositories (e.g., NCBI SRA [14]) or microbiome-specific databases (e.g., MG-RAST [15] and EBI Metagenomics [16]). To support large-scale mining of the existing microbiome big data, tools have been introduced to organize metagenomes with unified sequence processing standard operating procedures (SOPs) (17), e.g., Qiita (18), gcMeta (19), and GMrepo (20). These tools typically support queries based on taxonomy terms (e.g., species name), sequence fragments, or Structured Query Language (SQL)-like metadata. To support the search of newly generated data sets against the existing microbiome big data based on taxonomic or functional similarity, Microbiome Search Engine (MSE) (21) was recently introduced, and it shows promise for search-based multiple-disease classification in a cross-cohort, sequence-platform-insensitive, and contamination-tolerant manner (22). However, it supports only amplicon sequencing-based data sets, which limits the queries to those probing taxonomical similarity of microbiomes (21).

**FIG 1:**
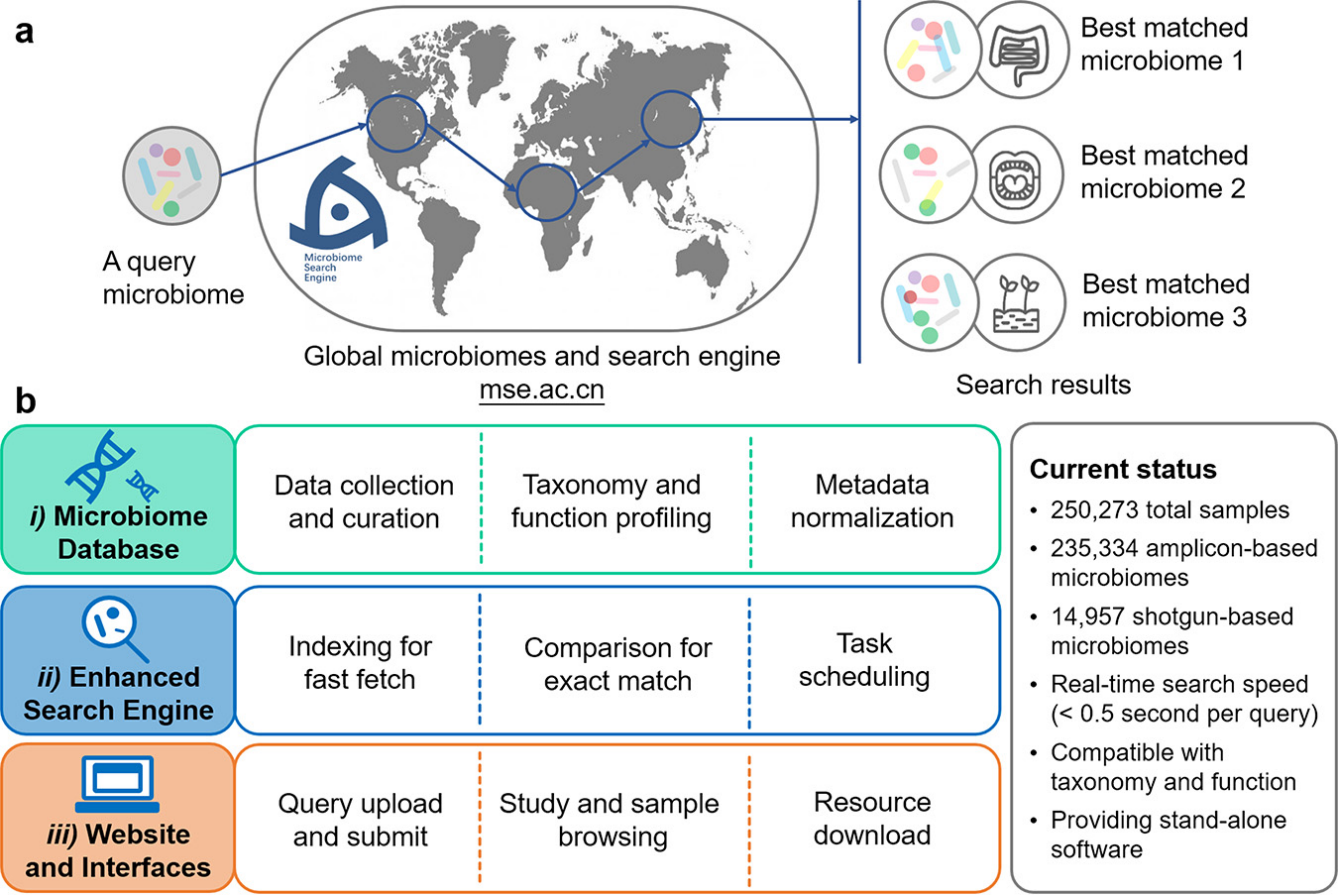
Design principles of MSE 2. (a) MSE 2 enables the search of a given amplicon- or shotgun-based microbiome against a large database, based on the whole-microbiome-level taxonomical or functional similarity between microbiomes. (b) The three key components of MSE 2 include a well-organized database, an enhanced search engine, and a Web-based interface.

To address this limitation, here we introduce Microbiome Search Engine 2 (http://mse.ac.cn), which enables the search of an amplicon or a shotgun WGS-based query microbiome against a large database based on the “functional” similarity of the microbiome (“taxonomical” similarity is also supported) (Fig. 1a). This platform, a significant improvement over the previous version (21), consists of three main components (Fig. 1b): (i) a well-maintained and regularly updated microbiome database that has been expanding since 2016 (see Fig. S1 in the supplemental material) and currently contains over 250,000 globally sampled (human, animal, marine, soil, etc.), curated microbiomes (both WGS- and amplicon-based samples) that are associated with a unified scheme of metadata from 798 studies; (ii) an enhanced search engine kernel that is compatible with both amplicon and shotgun WGS-based sequences and enables real-time searches against the database for best matches in microbiome taxonomy or function; and (iii) a Web-based graphical user interface that provides easy-use searching, data browsing, and tutoring.

10.1128/mSystems.00943-20.1 FIG S1 The MSE 2 microbiome database has been regularly maintained and updated since 2016. Download FIG S1, PDF file, 0.08 MB. Copyright © 2021 Jing et al. 2021 Jing et al. This content is distributed under the terms of the Creative Commons Attribution 4.0 International license.

## RESULTS

### Microbiome database. (i) Data collection and curation.

Clean sequences (refer to Materials and Methods) and their metadata were collected mainly from the Qiita (18), EBI (16), SRA (14), and MG-RAST (15) repositories. The common items of metadata for each study (e.g., project name, description, publication, etc.) (Table 1, project metadata) and sample (e.g., habitat, sequencing type, sampling year, etc.) (Table 1, sample metadata) were selected and manually integrated into a specific format, while the complete original metadata were also preserved. To ensure technical comparability and searchability among microbiome samples, sequences were preprocessed and profiled by unified methods according to sequence types (i.e., amplicon based or shotgun WGS based [Table 2]; for details, see Materials and Methods).

**TABLE 1:** Normalized metadata format of the MSE 2 database

Metadata	Content
Project metadata	
Project ID	The unique identifier of this project/study in the MSE 2 database
Project title	The project name and description in the MSE 2 database
Institute	The original producer of the study
Principal investigator	The original principal investigator of the study
Publication title	The paper’s title of the study, if applicable
Publication journal	The journal’s name of the paper, if applicable
Source	Link to the data source page, if applicable
Sequence type	Sequence type, 16S rRNA gene amplicon, and/or WGS
Date	Publishing date of the study/paper

Sample metadata	
Sample metadata	Content
Project ID	The project ID to which the sample belonged
Sample ID	The unique ID of this sample in the MSE 2 database, initialed by its project ID
Habitat domain	Unified into 3 categories: human associated, animal associated, and environment
Habitat type	Unified into 22 categories (Table S1) to further explain the habitat domain
Habitat details	Detailed information of the habitat
Sampling site	Detailed sampling site information
Sampling product	The sampling material and product
Date	Sampling/publishing date of the sample
Country/region	Sampling country/region of the sample
Gender	Host gender of a human-associated habitat sample, if applicable
Age	Host age of a human-associated habitat sample, if applicable
Description	Additional description of sampling information, e.g., host health status, etc.
Amplicon sequence type	Amplicon marker and region, if applicable, e.g., 16S V4
Amplicon sequencing platform	Sequencing platform for amplicon sequences, if applicable
WGS	If the sample has WGS shotgun sequences
WGS platform	Sequencing platform for WGS, if applicable
Function	If the sample has functional annotation
NSTI	The nearest sequenced taxon index to quantify the accuracy of function profiles predicted from amplicons

**TABLE 2:** Configuration of MSE 2 for each sequence type and recommended software for preprocessing

Search type	Applicable sequence type	Recommended software(s) for sequence preprocessing (reference)	Similarity metrics
By OTU	16S rRNA gene amplicon	Parallel-META 3 (29), QIIME (35)	Meta-Storms (33)
By species	Shotgun WGS	MetaPhlAn 2 (25)	Dynamic Meta-Storms (34)
By function	16S rRNA gene amplicon and shotgun WGS	Parallel-META 3 for a 16S rRNA gene amplicon, integrated with a C++ implantation of PICRUSt 2 (32); HUMAnN 2 for shotgun WGS (26)	Bray-Curtis

10.1128/mSystems.00943-20.2 TABLE S1 Unified metadata categories and sample numbers from the MSE 2 microbiome database. Download Table S1, PDF file, 0.1 MB. Copyright © 2021 Jing et al. 2021 Jing et al. This content is distributed under the terms of the Creative Commons Attribution 4.0 International license.

### (ii) Database statistics.

**FIG 2:**
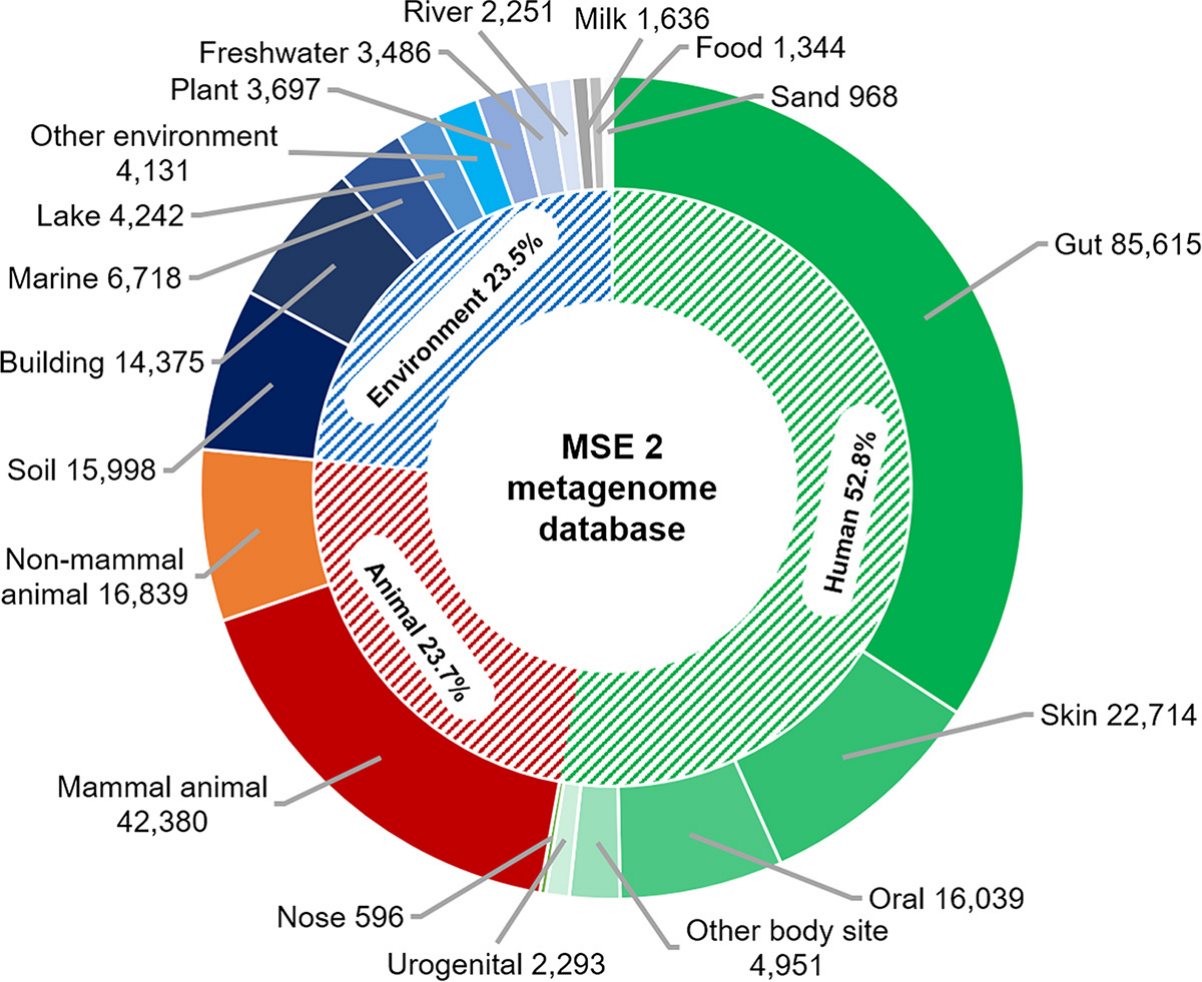
Distribution of sampling source in the microbiome database of MSE 2.

After the data preprocessing and curation (details are in Materials and Methods), a total of 250,273 microbiome samples from 798 projects/studies were included in the current MSE 2 database, including 14,957 shotgun WGS-based metagenomes and 235,334 16S rRNA gene amplicons. In terms of sampling source distribution (Fig. 2), human-associated habitats are the most frequent (52.8% in total; gut, 34.2%; skin, 9.1%; oral, 6.4%, etc.), followed by animal-associated habitats (23.7%), soil (6.4%), indoor environments (5.7%), and marine environments (2.7%) (for details, see Table S1 in the supplemental material).

### (iii) Database organization and management.

**FIG 3:**
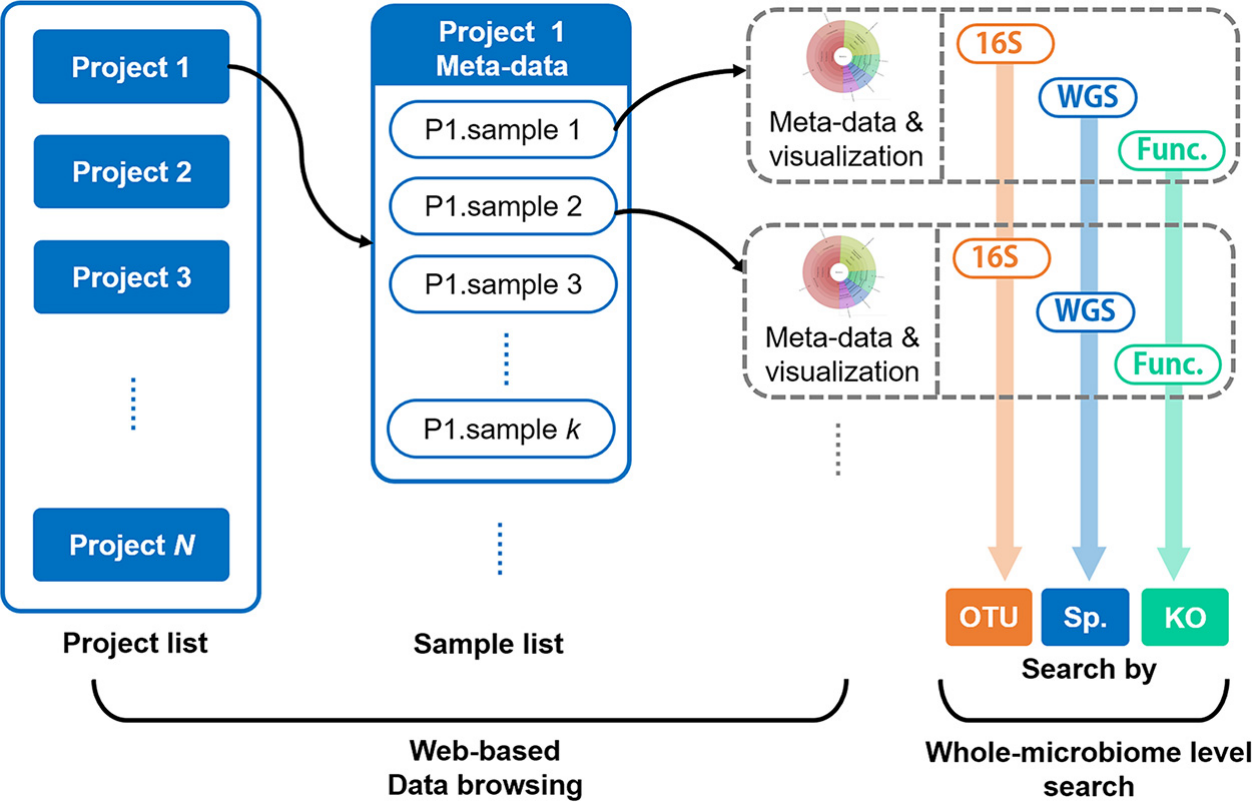
Database organization and structure in MSE 2. All microbiome samples were organized in two dimensions. For Web-based data browsing, samples were arranged by their studies and can be filtered via the various metadata. For whole-microbiome-level searches, samples were organized by compositional features (of either taxonomical or functional [Func.] profiles).

All microbiome samples are organized into two dimensions (Fig. 3). For Web-based data browsing (refer to the “Data browsing and download” section below), samples are arranged by studies and can be selected and filtered by the various metadata (e.g., habitat, sequence type, year, etc.). For searches based on taxonomical or functional similarity with microbiomes, samples were presorted by compositional features (e.g., operational taxonomic unit [OTU], species, or KEGG Orthology [KO] identifier [ID]) for indexing and searching (refer to the “Enhanced microbiome search engine” section below for details).

### Enhanced microbiome search engine. (i) Whole-microbiome-level search.

**FIG 4:**
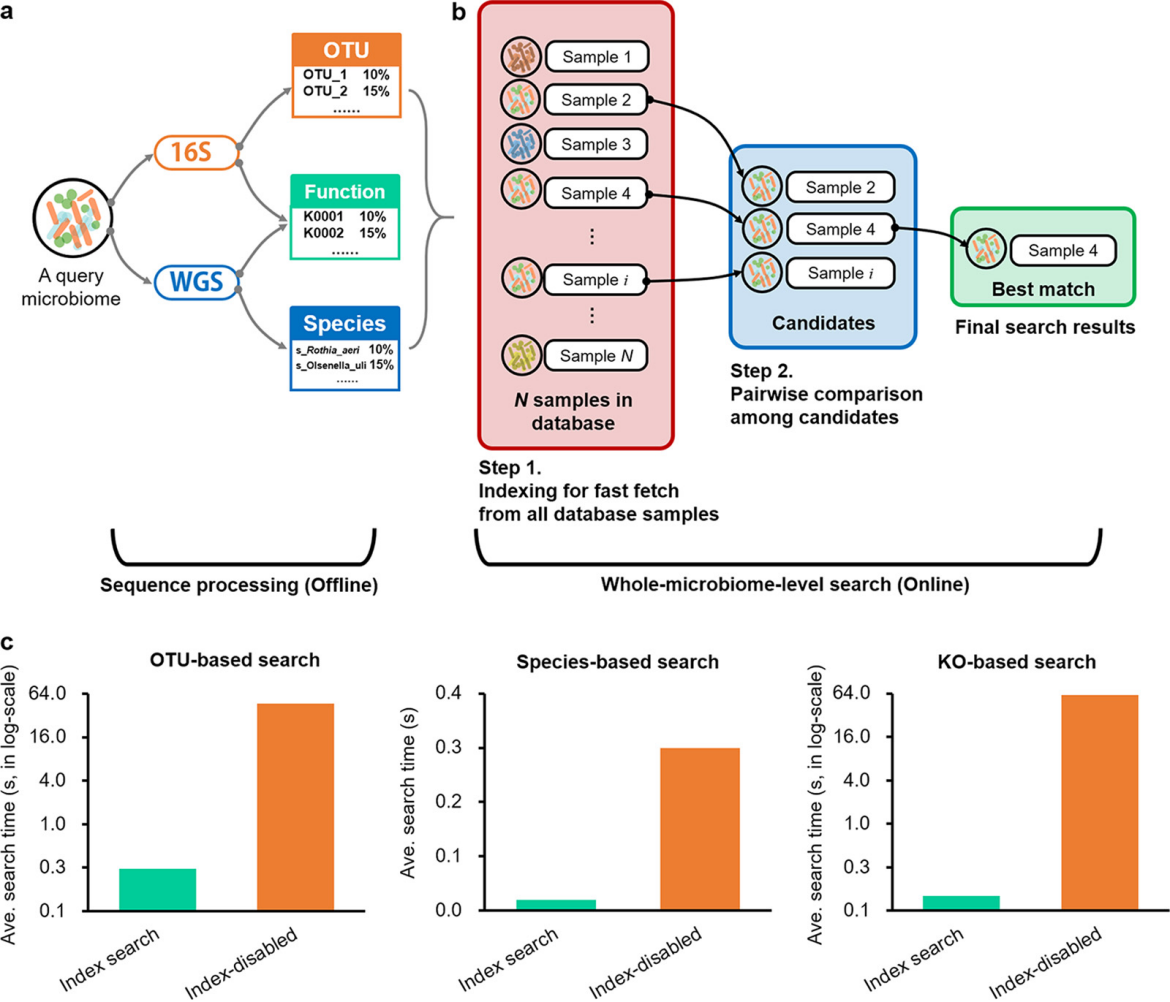
Workflow and performance of the search engine kernel. (a) Offline sequence preprocessing parses the compositional features of the query microbiome from sequences. (b) Two-tier indexing search to find the best-matched samples from the database with the highest similarity to the query. (c) Comparison of the average running times between index searching and index-disabled exhaustive searching for each search type.

The search engine, as the kernel of MSE 2, was developed by C++ and optimized by OpenMP-based parallel computing. With a given query microbiome, MSE 2 searches it against the entire microbiome database for best-matched samples that have the highest taxonomical or functional similarity. The search results present the taxonomical or functional profiles of the matches, the quantitative similarity values (refer to Materials and Methods for more details) compared to the query, and their metadata information (refer to the “Microbiome search and interpretation of the search results” section below for more details). Compared to the previous version (21), which accepts only OTUs from 16S rRNA gene amplicons as the query, the search engine extended its capability by supporting OTU-based (via profiles derived from 16S rRNA gene amplicons), species-based (via profiles derived from shotgun WGS) searches, and metabolic function-based (via profiles derived from either shotgun WGS or 16S rRNA gene amplicons) searches (Fig. 4a).

### (ii) Speed and scheduling.

Benefited by a two-tier indexing and searching strategy (Fig. 4b; refer to Materials and Methods for details), this search engine is typically 1 to 2 orders of magnitude faster than exhaustive searches that directly compare the query to all the database samples. To test the indexing efficiency and searching speed of MSE 2, we performed OTU-based, species-based, and function-based searches against the entire database and compared the search time to that of an index-disabled exhaustive search (the exhaustive search is for in-house performance evaluation only and not provided in the public online service of MSE 2). Each process was repeated 10 times, and only the search running times (excluding the upload time, visualization time, and Web page loading time to avoid potential bias caused by system and network latency) were recoded and compared. The results showed that the indexing strategy accelerates the search speeds by up to 193 times, 15 times, and 605 times, respectively, for OTU-based, species-based, and KO ID-based searches (Fig. 4c and Table 3), corresponding to a real-time response of within 0.5 s for a whole-microbiome-level query against the over 250,000 samples. In addition, the online search service follows the “first come, first served” principle implemented by queue-based task scheduling, so that the computing resources are utilized efficiently.

**TABLE 3:** Performance of index-based searches and index-disabled exhaustive searches^*a*^

Search type	Index-based search time (s)	Index-disabled search time (s)	Avg speedup
By OTU	0.241 ± 0.004	46.524 ± 1.029	193.345
By species	0.020 ± 0.001	0.300 ± 0.035	14.883
By function	0.101 ± 0.002	60.886 ± 0.189	605.076

a Each search procedure was repeated 10 times.

### Graphical Web-based portal. (i) Web-based user interface.

**FIG 5:**
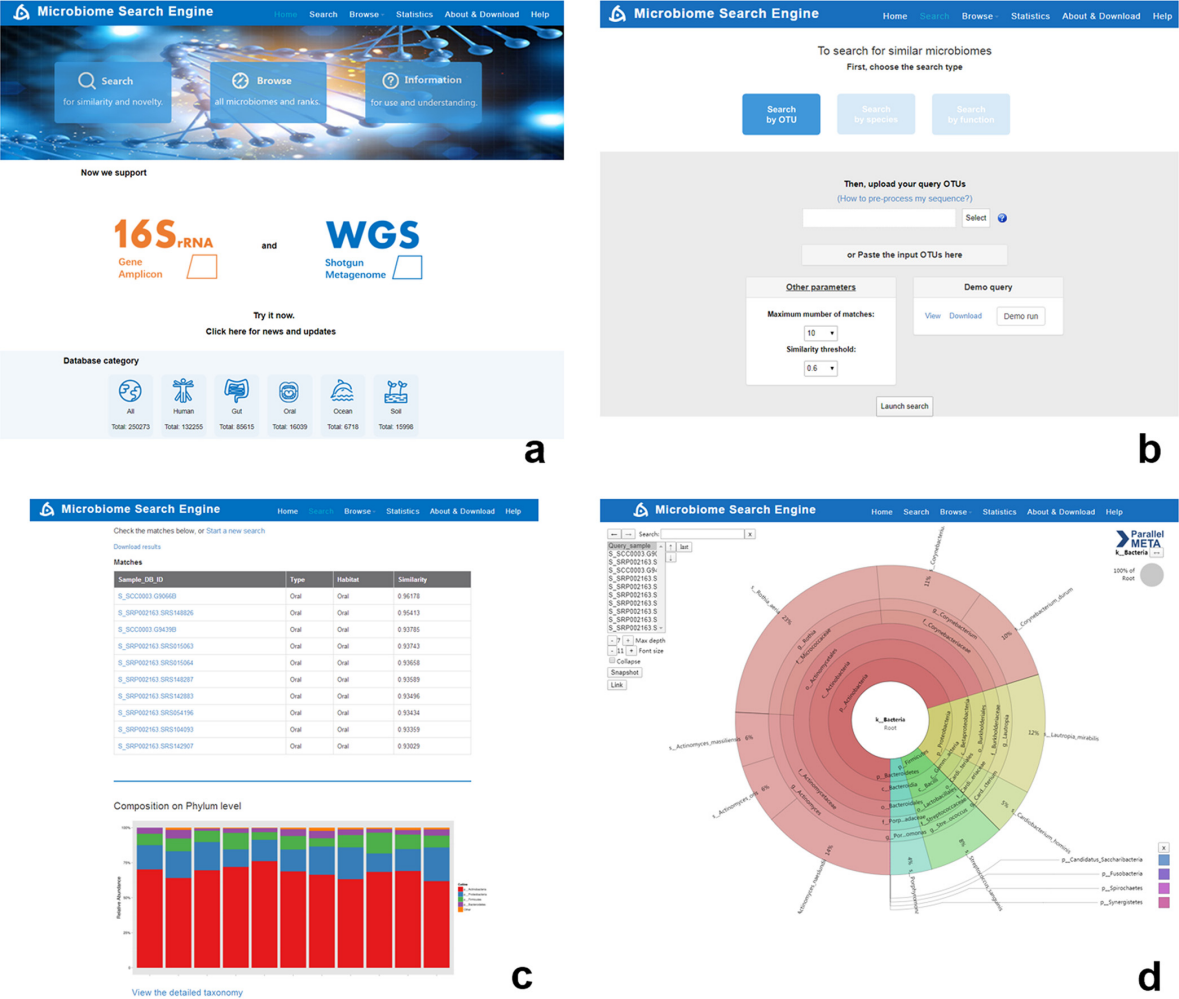
Screenshots of the MSE 2 website. (a) Homepage of MSE 2 with quick-use links and basic statistical information from the database. (b) The search page enables two forms of input (by file upload or content paste) and provides demo runs. (c) Result page of an example WGS query by a species-based search. (d) Interactive visualization of a search result that compares the taxonomies of the query and matches, which can be accessed via the link at the bottom of result page in panel c.

MSE 2 is freely accessible via http://mse.ac.cn via Web browsers. Developed by PHP and MySQL under a Linux server, this website provides a user-friendly graphical interface (Fig. 5) for searching, data browsing, and data uploading/downloading. Tutorial materials are available for users to adjust the parameters for customized functions and result interpretation. Notifications of database updates, system maintenance, and other related information are regularly published. Users can also post any questions or bugs at the Help Desk and obtain replies via e-mail.

### (ii) Microbiome search and interpretation of the search results.

For microbiome searches, MSE 2 accepts the compositional features of a sample (OTUs, species, or KO IDs) as queries. Notably, query microbiomes should be preprocessed from sequences into compositional features in an way identical to that used for the database samples. Table 2 summarizes the recommended software for sequence processing for each sequence type, and the detailed analytical protocol is available via the “Search” or “Help” page. To submit a search, users first choose a search type from “Search by OTU,” “Search by species,” and “Search by function,” depending on the type of query input (Fig. 5b). Then the query can be either uploaded from a tabular plain-text file or directly pasted into the text box of the Web page. Users can also specify other parameters, such as the maximum match number (the default is 10) and the cutoff similarity (the default is 0.6).

In the result page (Fig. 5c), top-matched samples from the database are listed with sample IDs, habitats, and similarity values relative to the query (nearest sequenced taxon index [NSTI] values are also provided for 16S rRNA gene-inferred functional profiles). Each sample ID is linked to its corresponding page with detailed full metadata (e.g., source study, sampling site, sequence type, etc.). The microbial compositions of the query and matched samples are visualized via both the bar chart and the Krona-based (23) interactive animation (Fig. 5d), so as to illustrate their links and distinctions in detail. Furthermore, all the above search results are packed for download in the result page for subsequent in-depth meta-analysis and data mining by users.

### (iii) Data browsing and download.

The MSE 2 online service provides two ways of sample browsing.

*(a) Browse by project.* In the project list page, samples are organized per project, and all projects are listed and sorted by project ID. Project pages can be accessed by clicking the project ID in the list or searched by metadata key words. Each project page contains the unified metadata (e.g., study title, publication, etc.) (Table 1, project metadata), original full list of metadata, links to samples in this project, and links to its data source.

*(b) Browse by sample.* In the sample list page, all samples are listed and sorted by sample ID, and samples can also be selected by a metadata filter for specific habitat, sequencing type, sampling year, etc. For a given sample in the database, all the unified metadata information (Table 1, sample metadata) can be displayed and the microbial taxonomy hierarchy visualized by Krona (23) by clicking on the sample ID.

## DISCUSSION

In this work, we introduce Microbiome Search Engine 2 (MSE 2), which features (i) an expanded database of over 250,000 shotgun metagenomic and 16S rRNA gene amplicon samples associated with unified metadata collected from 798 studies and (ii) an enhanced search engine for real-time and fast (<0.5 s per query) searches for best-matched microbiomes via not just taxonomic but also functional profiles. The value of a search-based strategy has been demonstrated for defining the novelty of microbiome samples (21) and for cross-cohort disease diagnosis (22, 24). By adding a function-based dimension for these and related applications, MSE 2 should accelerate large-scale mining of the ever-expanding metagenome data space.

## MATERIALS AND METHODS

### Sequence preprocessing of the microbiome database.

For shotgun sequences, MetaPhlAn2 (25) was used for species-level bacterial taxonomy assignment, and functional profiles were analyzed by HUMAnN2 (26) using “uniref90 gene families” and annotated on the basis of the KEGG Orthology (KO) database (27). For 16S rRNA gene amplicon sequences, OTUs were picked against Greengenes reference data (version 13-8) (28) with 97% similarity by Parallel-META 3 (29), and the relative abundances of OTUs were corrected by amplicon copy numbers that were parsed from the IMG/M database (30). Then the functional profiles were translated into KEGG orthologs by PICRUSt 2 (31, 32), while the NSTI (nearest sequenced taxon index) values that measure the prediction reliability were also recorded.

### Curation of the microbiome database.

All the data sets in the MSE 2 microbiome database are “clean” DNA sequence reads, i.e., reads that have already passed the initial sequence quality control process, including primer and tag clipping, host DNA removal, filtering of low-quality reads, etc. Microbiome samples were further curated based on the comprehensiveness of metadata and the quality of taxonomical or functional profiling. Specifically, samples that lack source habitat information were excluded (e.g., the habitat domain, habitat type, or habitat details in Table 1). Moreover, those amplicon samples with either <500 total reads or >20% reads that cannot be annotated were removed from the microbiome database.

### Indexing strategy of the microbiome search engine.

MSE 2 performs a two-tier indexing strategy (21) in order to enable real-time speed searches of large microbiome databases (Fig. 4b). To build the database index, for each database entry sample, MSE 2 partitions its profile features so that the compositional complexity is reduced. For taxonomic profiles (OTUs and species), features are sorted and merged by family-level taxa; for functional profiles, KEGG orthologs that belong to the same KEGG BRITE level 2 pathways are combined. The merged features are treated as the index keys. When a given query microbiome is searched, in step 1, the search engine parses its index keys in the same way as the database entries and dynamically selects those “candidate matches” with the shortest distances to the query sample on the index keys. Then, in step 2, MSE 2 identifies the top matches via a pairwise comparison between the query and each of the “candidate matches” using the similarity algorithms (refer to the “Similarity algorithms of the microbiome search engine” section below for details). Since the index keys are from a particular partition of the profile features, this two-tier search is typically 1 to 2 orders of magnitude faster than exhaustive searches that directly compare the query to all the database samples (refer to the “Speed and scheduling” section above for details).

### Similarity algorithms of the microbiome search engine.

After indexing, MSE 2 calculates the whole-microbiome-level similarity (in taxonomy or function) between the query and each candidate selected by indexing. For OTU- and species-based searches, MSE 2 uses the Meta-Storms (33) and Dynamic Meta-Storms (34) algorithms, respectively, both of which employ phylogeny-based metrics to quantitatively assess the similarity between microbiome samples. For function-based searches, MSE 2 applies Bray-Curtis dissimilarity to compare query and database entries based on relative abundances of KEGG orthologs.

### Implementation of the MSE 2 system and the Web-based portal.

MSE 2 runs under a CentOS Linux (release version 7.4) operating system. The search engine, as the kernel of MSE 2, was implemented by C++ and optimized by OpenMP-based parallel computing. The online service system was designed and constructed based on the LAMP (consisted of Linux, Apache, MySQL, and PHP) architecture. In this system, Apache software provided the accessibility of the Web pages that were developed with the PHP language. The metadata of projects and samples were arranged using the MySQL database engine. All data of MSE 2 were stored in a RAID 5 (Redundant Arrays of Independent Disks 5) storage node for data safety and security, while the index of the search engine was kept on a solid-state drive (SSD) RAID for fast fetch.

### Source code and data availability.

The microbiome database, including microbiome taxonomy, function, and metadata, are open for download from the MSE 2 website. The kernel search engine software was released on GitHub (https://github.com/qibebt-bioinfo/meta-storms) for standalone use with user-defined microbiome databases.

## References

[1] Fierer N, Jackson RB. 2006. The diversity and biogeography of soil bacterial communities. Proc Natl Acad Sci U S A 103:626–631. doi:10.1073/pnas.0507535103. 16407148 PMC1334650

[2] Steele JA, Countway PD, Xia L, Vigil PD, Beman JM, Kim DY, Chow CE, Sachdeva R, Jones AC, Schwalbach MS, Rose JM, Hewson I, Patel A, Sun F, Caron DA, Fuhrman JA. 2011. Marine bacterial, archaeal and protistan association networks reveal ecological linkages. ISME J 5:1414–1425. doi:10.1038/ismej.2011.24. 21430787 PMC3160682

[3] Qin N, Yang F, Li A, Prifti E, Chen Y, Shao L, Guo J, Le Chatelier E, Yao J, Wu L, Zhou J, Ni S, Liu L, Pons N, Batto JM, Kennedy SP, Leonard P, Yuan C, Ding W, Chen Y, Hu X, Zheng B, Qian G, Xu W, Ehrlich SD, Zheng S, Li L. 2014. Alterations of the human gut microbiome in liver cirrhosis. Nature 513:59–64. doi:10.1038/nature13568. 25079328

[4] Halfvarson J, Brislawn CJ, Lamendella R, Vazquez-Baeza Y, Walters WA, Bramer LM, D'Amato M, Bonfiglio F, McDonald D, Gonzalez A, McClure EE, Dunklebarger MF, Knight R, Jansson JK. 2017. Dynamics of the human gut microbiome in inflammatory bowel disease. Nat Microbiol 2:17004. doi:10.1038/nmicrobiol.2017.4. 28191884 PMC5319707

[5] Franzosa EA, Sirota-Madi A, Avila-Pacheco J, Fornelos N, Haiser HJ, Reinker S, Vatanen T, Hall AB, Mallick H, McIver LJ, Sauk JS, Wilson RG, Stevens BW, Scott JM, Pierce K, Deik AA, Bullock K, Imhann F, Porter JA, Zhernakova A, Fu J, Weersma RK, Wijmenga C, Clish CB, Vlamakis H, Huttenhower C, Xavier RJ. 2019. Gut microbiome structure and metabolic activity in inflammatory bowel disease. Nat Microbiol 4:293–305. doi:10.1038/s41564-018-0306-4. 30531976 PMC6342642

[6] Zeller G, Tap J, Voigt AY, Sunagawa S, Kultima JR, Costea PI, Amiot A, Bohm J, Brunetti F, Habermann N, Hercog R, Koch M, Luciani A, Mende DR, Schneider MA, Schrotz-King P, Tournigand C, Tran Van Nhieu J, Yamada T, Zimmermann J, Benes V, Kloor M, Ulrich CM, von Knebel Doeberitz M, Sobhani I, Bork P. 2014. Potential of fecal microbiota for early-stage detection of colorectal cancer. Mol Syst Biol 10:766. doi:10.15252/msb.20145645. 25432777 PMC4299606

[7] Teng F, Yang F, Huang S, Bo CP, Xu ZZ, Amir A, Knight R, Ling JQ, Xu J. 2015. Prediction of early childhood caries via spatial-temporal variations of oral microbiota. Cell Host Microbe 18:296–306. doi:10.1016/j.chom.2015.08.005. 26355216

[8] Forslund K, Hildebrand F, Nielsen T, Falony G, Le Chatelier E, Sunagawa S, Prifti E, Vieira-Silva S, Gudmundsdottir V, Pedersen HK, Arumugam M, Kristiansen K, Voigt AY, Vestergaard H, Hercog R, Costea PI, Kultima JR, Li J, Jorgensen T, Levenez F, Dore J, MetaHIT consortium, Nielsen HB, Brunak S, Raes J, Hansen T, Wang J, Ehrlich SD, Bork P, Pedersen O. 2015. Disentangling type 2 diabetes and metformin treatment signatures in the human gut microbiota. Nature 528:262–266. doi:10.1038/nature15766. 26633628 PMC4681099

[9] Gopalakrishnan V, Spencer CN, Nezi L, Reuben A, Andrews MC, Karpinets TV, Prieto PA, Vicente D, Hoffman K, Wei SC, Cogdill AP, Zhao L, Hudgens CW, Hutchinson DS, Manzo T, Petaccia de Macedo M, Cotechini T, Kumar T, Chen WS, Reddy SM, Szczepaniak Sloane R, Galloway-Pena J, Jiang H, Chen PL, Shpall EJ, Rezvani K, Alousi AM, Chemaly RF, Shelburne S, Vence LM, Okhuysen PC, Jensen VB, Swennes AG, McAllister F, Marcelo Riquelme Sanchez E, Zhang Y, Le Chatelier E, Zitvogel L, Pons N, Austin-Breneman JL, Haydu LE, Burton EM, Gardner JM, Sirmans E, Hu J, Lazar AJ, Tsujikawa T, Diab A, Tawbi H, Glitza IC, . 2018. Gut microbiome modulates response to anti-PD-1 immunotherapy in melanoma patients. Science 359:97–103. doi:10.1126/science.aan4236. 29097493 PMC5827966

[10] Integrative HMP Research Network Consortium. 2019. The Integrative Human Microbiome Project. Nature 569:641–648. doi:10.1038/s41586-019-1238-8. 31142853 PMC6784865

[11] Thompson LR, Sanders JG, McDonald D, Amir A, Ladau J, Locey KJ, Prill RJ, Tripathi A, Gibbons SM, Ackermann G, Navas-Molina JA, Janssen S, Kopylova E, Vazquez-Baeza Y, Gonzalez A, Morton JT, Mirarab S, Zech Xu Z, Jiang L, Haroon MF, Kanbar J, Zhu Q, Jin Song S, Kosciolek T, Bokulich NA, Lefler J, Brislawn CJ, Humphrey G, Owens SM, Hampton-Marcell J, Berg-Lyons D, McKenzie V, Fierer N, Fuhrman JA, Clauset A, Stevens RL, Shade A, Pollard KS, Goodwin KD, Jansson JK, Gilbert JA, Knight R, Earth Microbiome Project Consortium. 2017. A communal catalogue reveals Earth's multiscale microbial diversity. Nature 551:457–463. doi:10.1038/nature24621. 29088705 PMC6192678

[12] McDonald D, Hyde E, Debelius JW, Morton JT, Gonzalez A, Ackermann G, Aksenov AA, Behsaz B, Brennan C, Chen Y, DeRight Goldasich L, Dorrestein PC, Dunn RR, Fahimipour AK, Gaffney J, Gilbert JA, Gogul G, Green JL, Hugenholtz P, Humphrey G, Huttenhower C, Jackson MA, Janssen S, Jeste DV, Jiang L, Kelley ST, Knights D, Kosciolek T, Ladau J, Leach J, Marotz C, Meleshko D, Melnik AV, Metcalf JL, Mohimani H, Montassier E, Navas-Molina J, Nguyen TT, Peddada S, Pevzner P, Pollard KS, Rahnavard G, Robbins-Pianka A, Sangwan N, Shorenstein J, Smarr L, Song SJ, Spector T, Swafford AD, Thackray VG, . 2018. American Gut: an open platform for citizen science microbiome research. mSystems 3:e00031-18. doi:10.1128/mSystems.00031-18. 29795809 PMC5954204

[13] Bork P, Bowler C, de Vargas C, Gorsky G, Karsenti E, Wincker P. 2015. Tara Oceans. Tara Oceans studies plankton at planetary scale. Introduction. Science 348:873. doi:10.1126/science.aac5605. 25999501

[14] Kodama Y, Shumway M, Leinonen R, International Nucleotide Sequence Database Collaboration. 2012. The Sequence Read Archive: explosive growth of sequencing data. Nucleic Acids Res 40:D54–D56. doi:10.1093/nar/gkr854. 22009675 PMC3245110

[15] Wilke A, Bischof J, Gerlach W, Glass E, Harrison T, Keegan KP, Paczian T, Trimble WL, Bagchi S, Grama A, Chaterji S, Meyer F. 2016. The MG-RAST metagenomics database and portal in 2015. Nucleic Acids Res 44:D590–D594. doi:10.1093/nar/gkv1322. 26656948 PMC4702923

[16] Mitchell AL, Scheremetjew M, Denise H, Potter S, Tarkowska A, Qureshi M, Salazar GA, Pesseat S, Boland MA, Hunter FMI, Ten Hoopen P, Alako B, Amid C, Wilkinson DJ, Curtis TP, Cochrane G, Finn RD. 2018. EBI Metagenomics in 2017: enriching the analysis of microbial communities, from sequence reads to assemblies. Nucleic Acids Res 46:D726–D735. doi:10.1093/nar/gkx967. 29069476 PMC5753268

[17] Santiago A, Panda S, Mengels G, Martinez X, Azpiroz F, Dore J, Guarner F, Manichanh C. 2014. Processing faecal samples: a step forward for standards in microbial community analysis. BMC Microbiol 14:112. doi:10.1186/1471-2180-14-112. 24884524 PMC4021188

[18] Gonzalez A, Navas-Molina JA, Kosciolek T, McDonald D, Vazquez-Baeza Y, Ackermann G, DeReus J, Janssen S, Swafford AD, Orchanian SB, Sanders JG, Shorenstein J, Holste H, Petrus S, Robbins-Pianka A, Brislawn CJ, Wang M, Rideout JR, Bolyen E, Dillon M, Caporaso JG, Dorrestein PC, Knight R. 2018. Qiita: rapid, web-enabled microbiome meta-analysis. Nat Methods 15:796–798. doi:10.1038/s41592-018-0141-9. 30275573 PMC6235622

[19] Shi W, Qi H, Sun Q, Fan G, Liu S, Wang J, Zhu B, Liu H, Zhao F, Wang X, Hu X, Li W, Liu J, Tian Y, Wu L, Ma J. 2019. gcMeta: a Global Catalogue of Metagenomics platform to support the archiving, standardization and analysis of microbiome data. Nucleic Acids Res 47:D637–D648. doi:10.1093/nar/gky1008. 30365027 PMC6324004

[20] Wu S, Sun C, Li Y, Wang T, Jia L, Lai S, Yang Y, Luo P, Dai D, Yang YQ, Luo Q, Gao NL, Ning K, He LJ, Zhao XM, Chen WH. 2019. GMrepo: a database of curated and consistently annotated human gut metagenomes. Nucleic Acids Res 48:D545–D553. doi:10.1093/nar/gkz764. PMC6943048 31504765

[21] Su X, Jing G, McDonald D, Wang H, Wang Z, Gonzalez A, Sun Z, Huang S, Navas J, Knight R, Xu J. 2018. Identifying and predicting novelty in microbiome studies. mBio 9:e02099-18. doi:10.1128/mBio.02099-18. 30425147 PMC6234870

[22] Su X, Jing G, Sun Z, Liu L, Xu Z, McDonald D, Wang Z, Wang H, Gonzalez A, Zhang Y, Huang S, Huttley G, Knight R, Xu J. 2020. Multiple-disease detection and classification across cohorts via microbiome search. mSystems 5:e00150-20. doi:10.1128/mSystems.00150-20. 32184368 PMC7380586

[23] Ondov BD, Bergman NH, Phillippy AM. 2011. Interactive metagenomic visualization in a Web browser. BMC Bioinformatics 12:385. doi:10.1186/1471-2105-12-385. 21961884 PMC3190407

[24] Su X, Jing G, Zhang Y, Wu S. 2020. Method development for cross-study microbiome data mining: challenges and opportunities. Comput Struct Biotechnol J 18:2075–2080. doi:10.1016/j.csbj.2020.07.020. 32802279 PMC7419250

[25] Truong DT, Franzosa EA, Tickle TL, Scholz M, Weingart G, Pasolli E, Tett A, Huttenhower C, Segata N. 2015. MetaPhlAn2 for enhanced metagenomic taxonomic profiling. Nat Methods 12:902–903. doi:10.1038/nmeth.3589. 26418763

[26] Franzosa EA, McIver LJ, Rahnavard G, Thompson LR, Schirmer M, Weingart G, Lipson KS, Knight R, Caporaso JG, Segata N, Huttenhower C. 2018. Species-level functional profiling of metagenomes and metatranscriptomes. Nat Methods 15:962–968. doi:10.1038/s41592-018-0176-y. 30377376 PMC6235447

[27] Kanehisa M, Goto S, Sato Y, Furumichi M, Tanabe M. 2012. KEGG for integration and interpretation of large-scale molecular data sets. Nucleic Acids Res 40:D109–D114. doi:10.1093/nar/gkr988. 22080510 PMC3245020

[28] McDonald D, Price MN, Goodrich J, Nawrocki EP, DeSantis TZ, Probst A, Andersen GL, Knight R, Hugenholtz P. 2012. An improved Greengenes taxonomy with explicit ranks for ecological and evolutionary analyses of bacteria and archaea. ISME J 6:610–618. doi:10.1038/ismej.2011.139. 22134646 PMC3280142

[29] Jing G, Sun Z, Wang H, Gong Y, Huang S, Ning K, Xu J, Su X. 2017. Parallel-META 3: comprehensive taxonomical and functional analysis platform for efficient comparison of microbial communities. Sci Rep 7:40371. doi:10.1038/srep40371. 28079128 PMC5227994

[30] Chen IA, Chu K, Palaniappan K, Pillay M, Ratner A, Huang J, Huntemann M, Varghese N, White JR, Seshadri R, Smirnova T, Kirton E, Jungbluth SP, Woyke T, Eloe-Fadrosh EA, Ivanova NN, Kyrpides NC. 2019. IMG/M v.5.0: an integrated data management and comparative analysis system for microbial genomes and microbiomes. Nucleic Acids Res 47:D666–D677. doi:10.1093/nar/gky901. 30289528 PMC6323987

[31] Langille MG, Zaneveld J, Caporaso JG, McDonald D, Knights D, Reyes JA, Clemente JC, Burkepile DE, Vega Thurber RL, Knight R, Beiko RG, Huttenhower C. 2013. Predictive functional profiling of microbial communities using 16S rRNA marker gene sequences. Nat Biotechnol 31:814–821. doi:10.1038/nbt.2676. 23975157 PMC3819121

[32] Douglas GM, Maffei VJ, Zaneveld JR, Yurgel SN, Brown JR, Taylor CM, Huttenhower C, Langille MGI. 2020. PICRUSt2 for prediction of metagenome functions. Nat Biotechnol 38:685–688. doi:10.1038/s41587-020-0548-6. 32483366 PMC7365738

[33] Su X, Wang X, Jing G, Ning K. 2014. GPU-Meta-Storms: computing the structure similarities among massive amount of microbial community samples using GPU. Bioinformatics 30:1031–1033. doi:10.1093/bioinformatics/btt736. 24363375

[34] Jing G, Zhang Y, Yang M, Liu L, Xu J, Su X. 2020. Dynamic Meta-Storms enables comprehensive taxonomic and phylogenetic comparison of shotgun metagenomes at the species level. Bioinformatics 36:2308–2310. doi:10.1093/bioinformatics/btz910. 31793979

[35] Caporaso JG, Kuczynski J, Stombaugh J, Bittinger K, Bushman FD, Costello EK, Fierer N, Pena AG, Goodrich JK, Gordon JI, Huttley GA, Kelley ST, Knights D, Koenig JE, Ley RE, Lozupone CA, McDonald D, Muegge BD, Pirrung M, Reeder J, Sevinsky JR, Turnbaugh PJ, Walters WA, Widmann J, Yatsunenko T, Zaneveld J, Knight R. 2010. QIIME allows analysis of high-throughput community sequencing data. Nat Methods 7:335–336. doi:10.1038/nmeth.f.303. 20383131 PMC3156573

