# Supplementary material for: Microbiome Search Engine 2: a Platform for Taxonomic and Functional Search of Global Microbiomes on the Whole-Microbiome Level
Source: mSystems. 2021 Jan 19;6(1):e00943-20. doi: 10.1128/mSystems.00943-20 (PMC7820668; doi:10.1128/mSystems.00943-20)
Supplement: FIG S1 [file mSystems.00943-20-sf001.pdf]

Supplementary figure

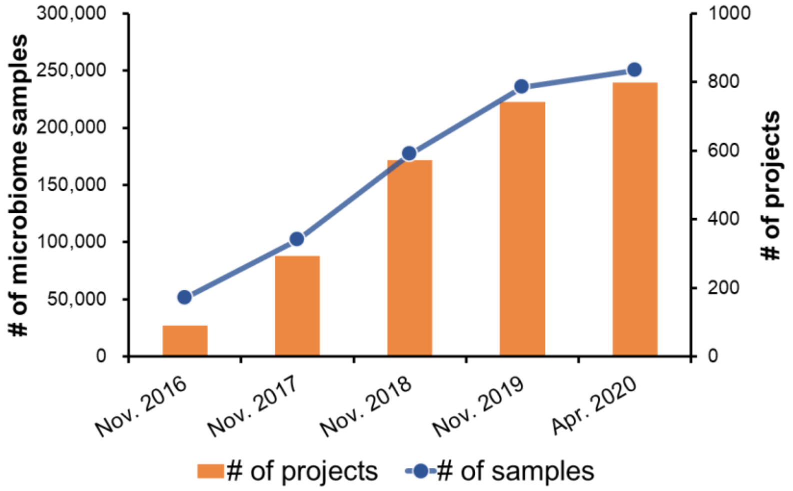

Fig. S1. MSE 2 microbiome database is regularly maintained and updated since 2016.
