# Supplementary material for: Microbiome Search Engine 2: a Platform for Taxonomic and Functional Search of Global Microbiomes on the Whole-Microbiome Level
Source: mSystems. 2021 Jan 19;6(1):e00943-20. doi: 10.1128/mSystems.00943-20 (PMC7820668; doi:10.1128/mSystems.00943-20)
Supplement: TABLE S1 [file mSystems.00943-20-st001.pdf]

**Supplementary table**

**Table S1. Unified metadata categories and sample numbers of MSE 2 microbiome database.**

| Habitat domain | Habitat type | # of samples | Rate |
|---|---|---|---|
| Human associated | Gut | 85,615 | 34.21% |
| Human associated | Skin | 22,714 | 9.08% |
| Human associated | Oral | 16,039 | 6.41% |
| Human associated | Other body site | 4,951 | 1.98% |
| Human associated | Urogenital | 2,293 | 0.92% |
| Human associated | Nose | 596 | 0.24% |
| Animal associated | Mammal animal | 42,380 | 16.93% |
| Animal associated | Non-mammal animal | 16,839 | 6.73% |
| Environment | Soil | 15,998 | 6.39% |
| Environment | Building | 14,375 | 5.74% |
| Environment | Marine | 6,718 | 2.68% |
| Environment | Lake | 4,242 | 1.69% |
| Environment | Other environment | 4,131 | 1.65% |
| Environment | Plant | 3,697 | 1.48% |
| Environment | Freshwater | 3,486 | 1.39% |
| Environment | River | 2,251 | 0.90% |
| Environment | Milk | 1,636 | 0.65% |
| Environment | Food | 1,344 | 0.54% |
| Environment | Sand | 968 | 0.39% |
